# A Treatise on Dislocations and Fractures of the Joints

**Published:** 1843-12-23

**Authors:** 


*A Treatise on Dislocations and Fractures of the Joints.* By Sir ASTLEY COOPER, Bart. F. R. S., etc. *A new Edition, much enlarged.* Edited by BRANSBY B. COOPER, F. R. S., Surgeon to Guy's Hospital. *With additional Observations, and a Memoir of the Author.* Philadelphia; Lea & Blanchard. 1844. 8vo. pp. 499.

A mere announcement of a new edition of this great work would be sufficient were it a mere reprint from the former one; but we are told by Mr. Bransby Cooper, in the Preface, that "the very grateful task of Editor" was assigned him by his uncle some time previous to his death, and that the present edition embodies much new matter, left by Sir Astley himself, together with additional cases, derived from the practice of the editor, and various other sources. Some condensation has been made in the original; this is generally slight and insignificant, although once or twice it leads to false inferences and mistakes. The work is a splendid monument to the genius of the author, and stands unrivalled as a monograph on the subject, but it is still the record of the opinions and practice of an individual, most distinguished it is true, but of an original and practical turn of mind, and not very erudite in the literature of the profession, who chose rather to state what he saw and knew, than to record the views of others. As Mr. B. Cooper undertook the duty of editor, he should have fulfilled it, and contrasted the opinions of Sir Astley Cooper with that of other distinguished surgeons of the day, "either to the confirmation or the condemnation of the views laid down by Sir Astley Cooper in the treatment of fractures and dislocations of the joints;" he should have placed the work on a level with the existing state of knowledge on the subject, and this he has not done. His opportunities and information should have qualified him for the task, and if his literary abilities were deficient, his notes might have been committed to other hands. Although an inefficient editor, Mr. Cooper's additions and comments are often valuable and judicious, although many are unimportant, and his style is generally careless, and sometimes obscure. No one was better qualified to supply the faults of omission on the part of the English editor than the eminent individual who has superintended the republication in this country, and has added numerous valuable cases. We regret that Dr. Warren did not embrace this opportunity of giving us his experience on this class of injuries, linking his name with that of his illustrious preceptor. The additional observations that he has favoured us with are detailed in the full spirit of the original, and are interesting and instructive. We will notice one point to which he has called attention, we believe for the first time—*the unfrequency of dislocation of the os femoris in the female.* Dr. Warren states that in an experience of thirty-eight years, he has never seen an instance of dislocation of the os femoris in an adult female, and one only in a female under age, a girl of nine years of age. Curious to discover if the experience of other surgeons accorded with his own, he made numerous inquiries during his visit to Europe in 1837 and 1838, which were generally received with surprise, the question apparently not having before been adverted to. For some time he could not ascertain a well authenticated case, until Mr. Clift, the Conservator of the Museum of the Royal College of Surgeons, London, mentioned one to him—a case of double dislocation into the foramina ovalia, which was never reduced. Subsequently he heard of a few cases, especially from Dr. Stevens of New York, who stated that he had seen two or three. Sir Astley Cooper mentions above fifty cases of dislocation in the male, and but one in an adult female, and one in a female under age. This accident must necessarily be exceedingly rare in the female, fracture of the neck of the femur, being frequent with them, and rare in the male. Can the accident peculiar to each be explained by any difference in the anatomical structure of the two sexes. Nine measurements of the circumference of the neck of the thigh bone in the male and female, showed "that the cervix of the os femoris in the female is less in circumference, by at least half an inch, than the same part in the male. This being the fact, a force applied to the femoris would be much more likely to produce fracture of the cervix in the female than in the male, and the comparative frequency of dislocation in the female would be necessarily diminished. Another fact which explains the more frequent occurrence of fractures of the cervix in the female, is, that the greater breadth of the pelvis in them produces a corresponding projection of the trochanter, which of course renders it more obnoxious to external violence," (p. 98.)

At p. 14 Sir Astley Cooper says, "I have read of dislocations of the hip in children, but their history is that of diseases of the hip joint, in which the dislocation has arisen from ulceration." A case is subsequently given, (p. 391, of dislocation of the femur on the dorsum ilii,) in a child seven years old, not seen by Sir Astley, but communicated to him by Mr. Daniell, a dresser in the hospital. Dr. Warren alludes to one incidentally in the paragraph quoted above, occurring in a female child nine years old. Should not Dr. Norris's case in a boy eleven years old have been mentioned, together with a few other authenticated cases on record, of dislocation of the femur in childhood?

The present edition is handsomely printed, and is illustrated with a beautiful engraving of the author. The wood cuts are indifferent.

